# Ribosomal protein L10 in mitochondria serves as a regulator for ROS level in pancreatic cancer cells

**DOI:** 10.1016/j.redox.2018.08.016

**Published:** 2018-08-24

**Authors:** Jun Yang, Zongmeng Chen, Nan Liu, Yijun Chen

**Affiliations:** State Key Laboratory of Natural Medicines and Laboratory of Chemical Biology, China Pharmaceutical University, 24 Tongjia St., Nanjing, Jiangsu Province 210009, People's Republic of China

**Keywords:** RPL10, ROS, Mitochondria, Regulation, Pancreatic cancer

## Abstract

Tumorigenesis is commonly known as a complicated process, in which reactive oxygen species (ROS) plays a critical role to involve in signal transduction, metabolism, cell proliferation and differentiation. Previously, ribosomal protein L10 (RPL10) was suggested to possess extra-ribosomal functions in pancreatic cancer cells in addition to being proposed as a tumor suppressor or transcription co-regulator. To better understand the relationship between RPL10 and tumorigenic potential in pancreatic cancer cells, chromatin immunoprecipitation sequencing reveals that RPL10 is unlikely to be a transcription factor without a specific binding motif for gene transcription. Additionally, transcriptome analysis indicates that RPL10 could regulate the expression of proteins related to ROS production. Moreover, RPL10 in mitochondria is closely associated with the regulation of ROS level by affecting Complex I activity and the subsequent events. Together, the present study suggests that the regulation of ROS level by mitochondrial RPL10 is one of the major extra-ribosomal functions in pancreatic cancer cells, which could be used as an indicator for the tumorigenesis of pancreatic cancer.

## Highlights

• RPL10 exists in mitochondria.
• RPL10 affects the expression of proteins related to oxidative stress and ROS generation.
• RPL10 regulates ROS level in pancreatic cancer cells.

## Graphical abstract

**Figure:**
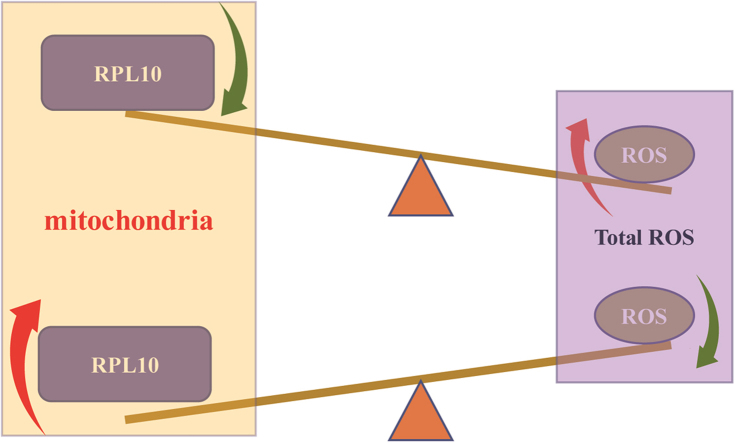

## 1 Introduction

Pancreatic cancer is known for its high morbidity and lethality with difficulty on diagnosis and therapy with 5-year survival rate of less than 5%. It is estimated that there are more than 54,000 new cases and 44,000 deaths related to pancreatic cancer in United States in 2018 [1]. Although there have been numerous efforts in developing new therapies for pancreatic cancer, little achievement has been made so far [2].

Human ribosomal protein L10 (RPL10), also known as QM, is a 25 kDa basic protein first found in nontumorigenic Wilms’ tumor, participating in the late steps of 60 S ribosome assembly [3], [4]. Despite its major roles in protein synthesis, many reports have indicated that RPL10 also possesses extra-ribosomal functions. Initially, when a cDNA/mRNA hybridization was performed between the closely paired nontumorigenic Wilms’ microcell hybrids and the tumorigenic Wilms’ patient containing the cDNA for RPL10 [3], RPL10 was suspected to involve in the maintenance of nontumorigenic state. Later, Jif-1 protein from chicken embryo fibroblasts was screened out to share 92% amino acid homology to RPL10 [5], confirming that Jif-1 could bind c-Jun, a member of transcription factor complex AP-1, and suggesting that Jif-1 is a negative regulator of c-Jun. Subsequently, the interaction between human RPL10 and c-Jun, a transcription factor in which zinc ion is indispensable for its DNA binding, further supported that RPL10 is a transcription regulatory factor related to c-Jun [6], [7]. This has driven the focus of RPL10 to cancer-related biological events in recent years. In early developmental stage of prostate cancer, RPL10 level was decreased, and higher level of RPL10 could promote tumor deterioration at late stage [8]. Significantly, through exome sequencing in T-cell acute lymphoblastic leukemia in 2012, an important mutation of RPL10 (R98S) was identified [9], and this mutation highly enhanced JAK-STAT signaling pathway [10] and affected the 60 S ribosome export adapter Nmd3 in the ribosomal P site in yeast [11]. Last year, it was reported that RPL10 level is higher in human epithelial ovarian cancer than normal ovarian tissues. At lower expression level of RPL10, cell viability, migration and invasion decreased while cell apoptosis increased. By contrast, when RPL10 was over-expressed, cell viability, migration and invasion increased while cell apoptosis decreased [12]. These phenomena have suggested that extra-ribosomal RPL10 could be an important player in cancer development. In our previous study, when dimethylamino parthenolide (DMAPT), a water-soluble analogue of parthenolide (PTL), was used as an affinity probe, RPL10 was identified as a direct target protein to affect cell proliferation in pancreatic cancer cell lines of PANC-1 and MIA PaCa-2, suggesting that RPL10 could be a novel target for the treatment of pancreatic cancer [13]. Consistent to our findings, it was recently reported that PTL directly binds RPL10 to inhibit Wnt/β-catenin signaling pathway through the down-regulation of transcription factors TCF/LEF1 in HEK293 cells [14], further highlighting the importance of RPL10 in the development of tumorigenesis. Although current evidence directs the roles of extra-ribosomal RPL10 to carcinogenesis and cancer development, how RPL10 functions its tumorigenic potential in pancreatic cancer cells remains largely unclear.

Meanwhile, rpl10 gene was discovered in diverse plant mitochondrial genomes [15], [16]. In human, a protein variant originated from RPL10 (H213Q) was found to associate with autism spectrum disorders, in which the expression of specific proteins was changed, including those involving in the redox-system [17]. Given the various extra-ribosomal functions of RPL10 and the necessary roles of mitochondria in the redox-system, it is natural and reasonable to explore whether RPL10 directly participates in the mitochondrial events in pancreatic cancer cells.

In the present study, we first utilized CHIP-Sequencing technique to clarify that RPL10 could not bind to specific promoter regions in PANC-1 cells. Then, we found that RPL10 can be localized in mitochondria to regulate ROS level through affecting mitochondrial Complex Ⅰ activity and cellular ATP production. Furthermore, Transcriptome-Sequencing analysis indicated that RPL10 in mitochondria participates in the regulation of ROS level by influencing the expression of proteins related to ROS generation. At the same time, alteration of RPL10 expression could affect ROS level in MIA PaCa-2 cells. Moreover, exogenous treatments of PANC-1 and MIAPaCa-2 cells by hydrogen peroxide (H_2_O_2_) and an antioxidant N-acetylcysteine (NAC) significantly altered the expression of RPL10. Together, the present study suggests that RPL10 could serve as a regulator in mitochondria to balance cellular ROS level during the process of tumor progression.

## 2 Results

### 2.1 RPL10 is unlikely to be a transcription factor

Given the properties of being a zinc finger protein [6] and the ability of binding c-Jun [7], we initially examined the possibility of RPL10 on gene transcription. After CHIP-Sequencing analysis from PANC-1 cells, 7603 peaks with an average 205 bp length on all chromosomes were identified as potential gene targets (Supplementary Fig. S1D). Based on E-value and binding sites, 6 binding motifs showed high scores (Supplementary Fig. S1A). Compared to classical transcription factors, approximately 2.7% binding sites are located upstream (~ 2000 bp) of the coding region in the chromatin without any promoters and 85.9% of the binding sites are found in intergenic and intron regions (Supplementary Fig. S1B). Interestingly, most binding sites of RPL10 are located on chromosome Y, but they are far from the coding regions. Collectively, all RPL10 binding sites on the chromosomes did not show any meaningful sites for the activation of gene transcription. To confirm the sequencing results, dual-luciferase reporter assay system was then used to determine whether RPL10 is involved in gene transcription. Genes with the highest scores, including fam213b, adamts16 and wt-1, were chosen to investigate the ability of RPL10 to bind their promoter regions respectively. Indeed, RPL10 was not able to activate the transcription of these genes (Supplementary Fig. S1C). In addition, the binding sites on c-jun gene were also analyzed to show undetectable signals from RPL10 binding (Supplementary Fig. S1E), suggesting that the binding between RPL10 and c-jun might be indirect. Therefore, different from previous prediction [7], RPL10 is unlikely to be a transcription factor for gene regulation.

### 2.2 RPL10 exists in mitochondria

**Fig. 1:**
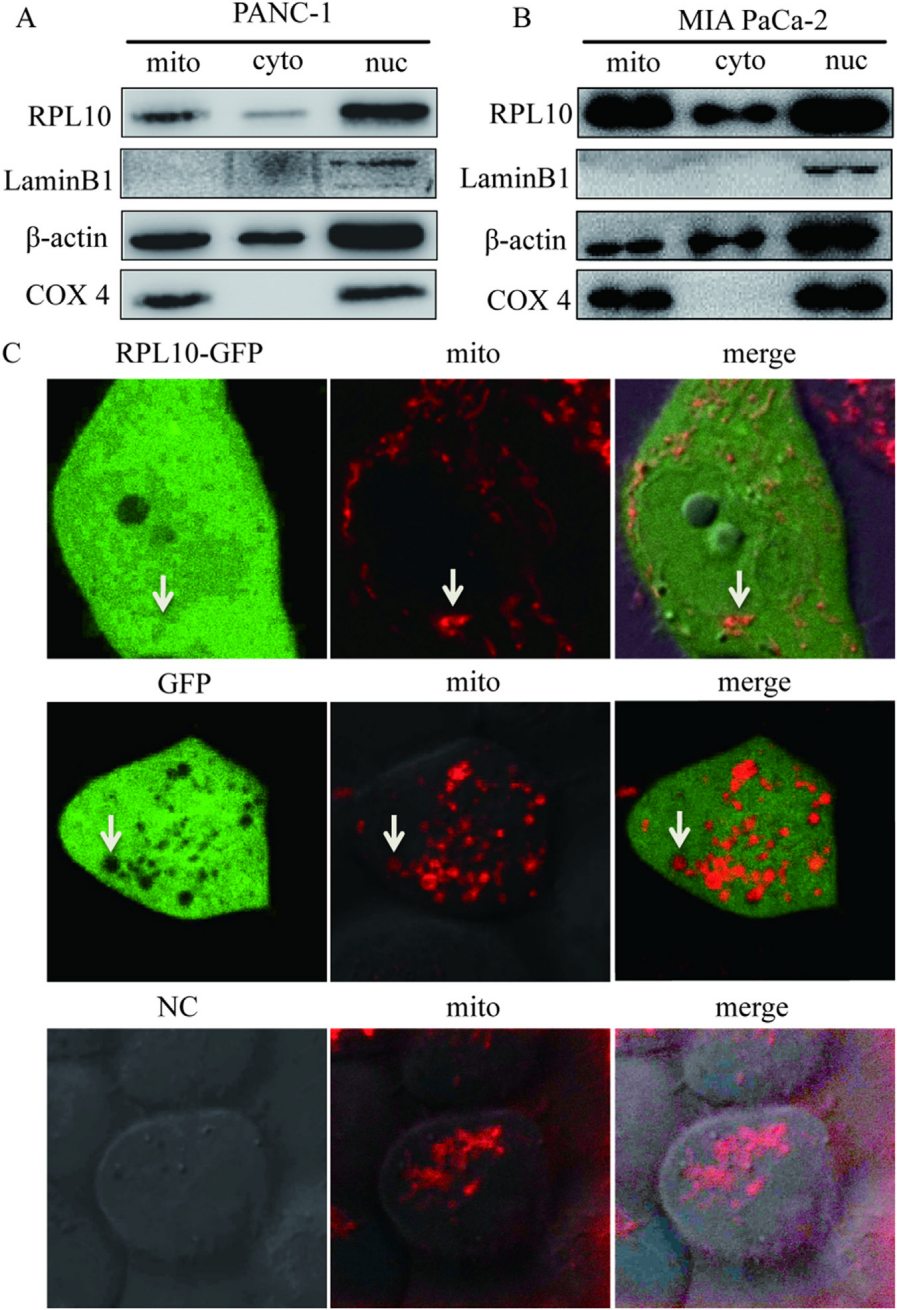
Mitochondrial localization of RPL10. (A) Detection of RPL10 in PANC-1 cells; (B) Detection of RPL10 in MIA PaCa-2 cells. In the Western blots, mito, cyto and nuc represent mitochondrial, cytoplasmic and nuclear extracts respectively. COX4 is a marker for mitochondrial proteins and LaminB1 is a marker for nuclear proteins. (C) Confocal images for RPL10 in mitochondria. RPL10-GFP fusion protein (upper panels) and GFP (middle panels) were detected with staining mitochondria by mitochondria dye, MitoTracker (mito). Lowest panels are blank control without GFP expression vector. White arrows indicate the speckles staining with MitoTracker fluorescence.

Mitochondrion has its own genome and ribosomal proteins (mitoribosome) for mitochondrial protein synthesis. Several mitoribosomal proteins have been reported to possess various functions in cellular processes in addition to the participation of protein synthesis [18], [19]. Within mitoribosomal proteins, mitochondrial ribosomal protein L10 (mRPL10) (GenBank:AAH52601.1), was reported to associate with Cyclin B1/Cdk1 activity and mitochondrial function [20]. Differently, RPL10 (GenBank:CAG33078.1) is identified as a nuclear ribosomal protein and is well recognized to exist in both nucleus and cytoplasm [21], [22]. However, prediction of subcellular localization of RPL10 using TargetP (https://omictools.com/targetp-tool) [23] and iPSORT (http://ipsort.hgc.jp/) resulted in a mitochondrial distribution. To examine whether RPL10 is able to enter to mitochondria, different portions of nuclear, mitochondrial and cytoplasmic proteins were individually isolated and compared from PANC-1 and MIA PaCa-2 cells. As shown in Fig. 1A and Fig. 1B, RPL10 could be obviously detected in the fraction of mitochondria in addition to nucleus and cytoplasm. To further confirm the mitochondrial localization of RPL10, a vector pd1EGFP-N1-rpl10 was constructed and used for the imaging of subcellular locations in PANC-1 cells (Fig. 1C). When only GFP was expressed, it was hardly seen green fluorescence in the mitochondria. On the other hand, in the presence of rpl10 in the expression vector, the cells were filled with green fluorescence, especially in mitochondria. Therefore, RPL10 is actually located in nucleus, cytoplasm as well as mitochondria, suggesting that RPL10 could be functionally related to mitochondria.

### 2.3 Knockdown of RPL10 reduced Complex Ⅰ activity and ATP production

**Fig. 2:**
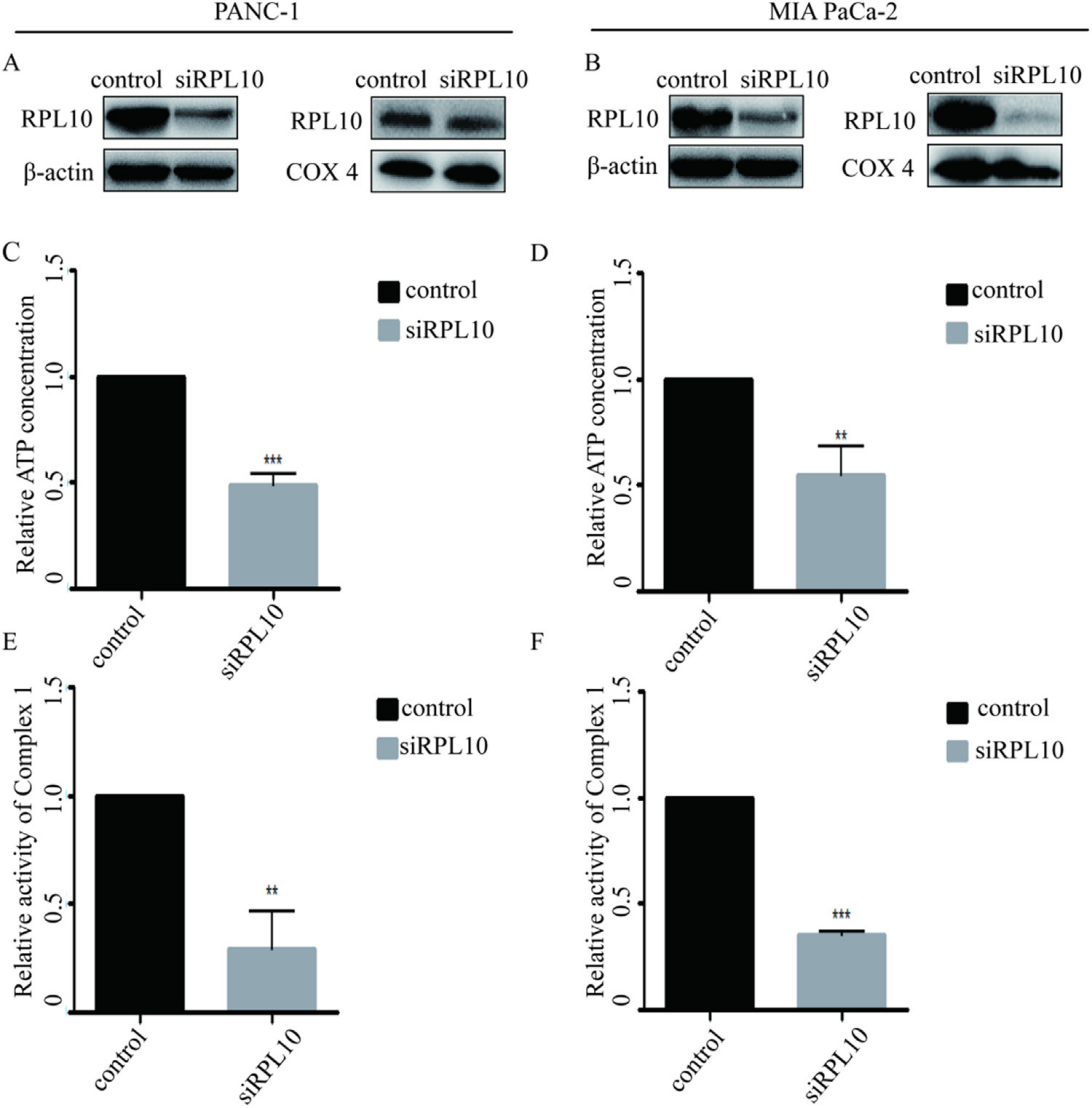
Reduction of ATP production and Complex Ⅰ activity by knock-down of RPL10. (A) RPL10 expression in PANC-1 cells; (B) RPL10 expression in MIA PaCa-2 cells. Total and mitochondrial RPL10 levels in normal cells (control) and knock-down of RPL10 by siRNA (siRPL10) were compared. (C) ATP concentration after knock-down of RPL10 in PANC-1 cells; (D) ATP concentration after knock-down of RPL10 in cells; (E) Relative activity of Complex Ⅰ after knock-down of RPL10 in PANC-1 cells; (F) Relative activity of Complex I after knock-down of RPL10 in MIA PaCa-2 cells. The ATP concentration and Complex Ⅰ activity in untreated cells are normalized as 1.0. Data show the average values with standard deviations from 3 independent experiments. * ** P < 0.001 and * * P < 0.01.

Mitochondrion is a critical organelle controlling a variety of important physiological processes, including energy supply, tricarboxylic acid cycle (TCA), fatty acid beta-oxidation and ROS production. To investigate the roles of RPL10 in mitochondria, knock-out of RPL10 was attempted using CRISPR/Cas9 technique in PANC-1 cells. However, due to the lethality to the cells, no positive clones were obtained from the knock-out. Consequently, knock-down of RPL10 with siRNA was subsequently performed in PANC-1 and MIA PaCa-2 cells. After knock-down of RPL10, the expression of RPL10 decreased in total lysates and mitochondrial proteins (Fig. 2A, B). At the same time, ATP concentration in PANC-1 and MIA PaCa-2 cells significantly decreased compared to the control (Fig. 2C, D), suggesting that their metabolic functions have been changed. It is well known that cancer cells have a higher demand for ATP and its precursors for biosynthesis, preferring using glycolytic pathway to consume glucose into lactate rather than completely oxidizing it at a high rate [24], [25]. Thus, the present results indicated that RPL10 has a close relationship with the energy usage and biological oxidation in cancer cells.

Meanwhile, the activity of mitochondrial Complex I-Ⅳ was examined after knocking-down RPL10. As anticipated, the activity of Complex Ⅰ significantly reduced from the knock-down of RPL10 in PANC-1 and MIA PaCa-2 cells (Fig. 2E, F), whereas no difference on the activity of Complex Ⅱ-Ⅳ was seen (Data not shown), indicating that RPL10 is most likely to involve in the function of mitochondrial Complex I. Given the fact that Complex Ⅰ catalyzes NADH oxidation and transports protons across the inner mitochondrial membrane, leading to energy and ROS production [26], [27], [28], [29], [30] and knock-down of RPL10 could result in the changes of Complex Ⅰ and ATP, it was crucial to investigate the relationship between RPL10 and ROS level in pancreatic cancer cells.

### 2.4 Reduction of RPL10 affected a variety of protein expression

**Fig. 3:**
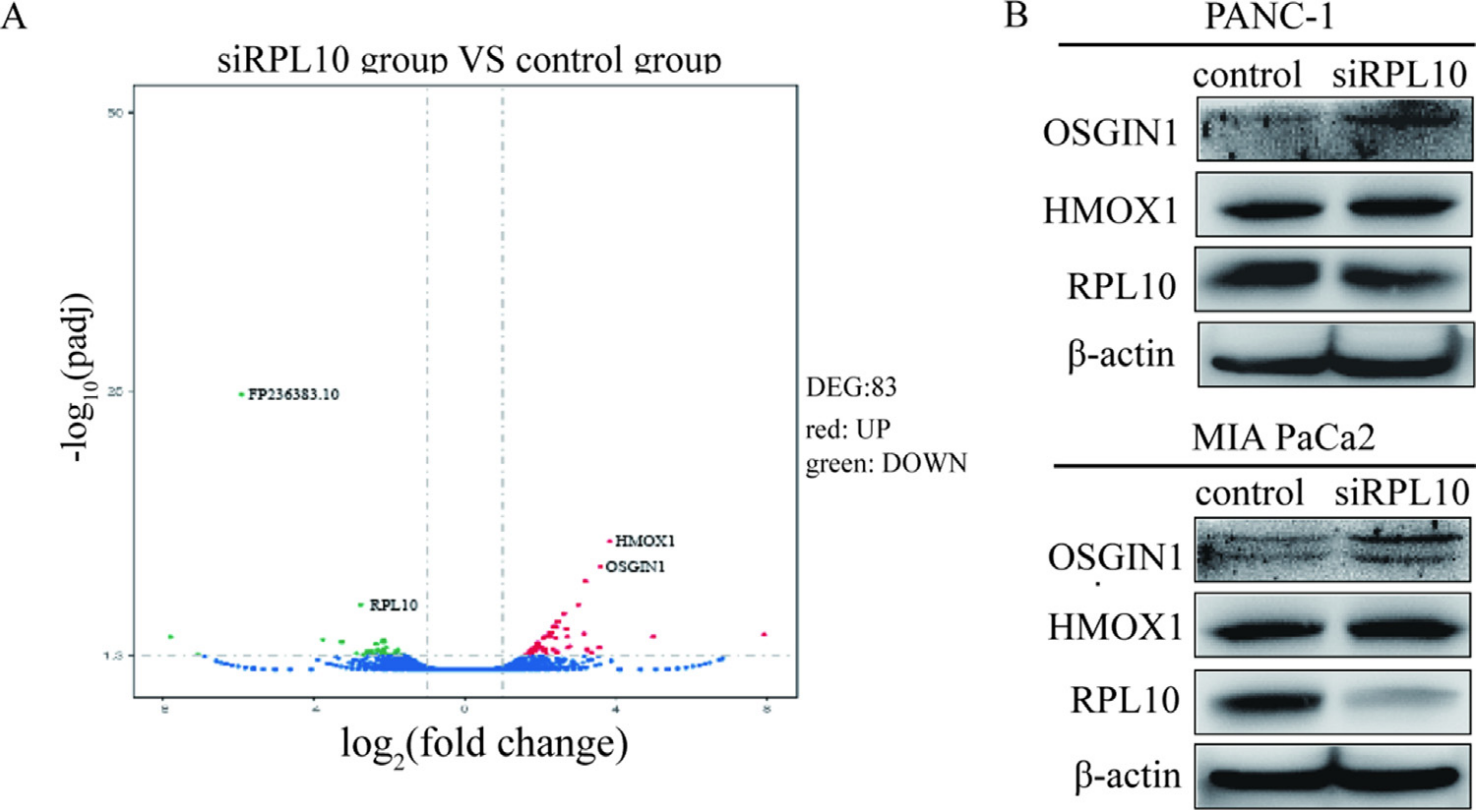
The differences of gene transcription and protein expression after knock-down of RPL10. (A) Volcano-plot of differential genes (DEG) from the transcriptome sequencing analysis. Red and green dots represent the genes up-regulated and down-regulated respectively. X-axis and Y-axis indicate the fold change of genes and *p*-value with adjustment (padj) of each gene respectively. (B) Western blots of OSGIN1 and HMOX1 expression after knock-down of RPL10 in PANC-1 and MIA PaCa-2 cells.

To identify gene transcription related to the expression or function of RPL10, a microarray-based transcriptome sequencing was conducted after RPL10 was knocked-down (Fig. 3A). The most significantly altered proteins are summarized in Supplementary Table 1. With the highest score, an unidentified gene FP236383.10 with a prediction of being a miRNA in the databases, decreased the most, which remains for further investigation. All other genes were remarkably up-regulated for their transcription, among which the proteins of osgin1 [31], [32], hmox1 [33], [34], atf3 [35], [36], spns2 [37], ppp1r15a [38] and tiparp [39] possess certain degree of relationship with redox homeostasis, suggesting that the change of RPL10 is closely associated with redox homeostasis in the cells. To verify such a relationship, two typical proteins involved in redox homeostasis, OSGIN1 and HMOX1, were chosen for experimental verification. When RPL10 was knocked-down in PANC-1 and MIA PaCa-2 cells, the expression of OSGIN1 indeed markedly increased, whereas HMOX1 showed little change in both cells (Fig. 3B), indicating that RPL10 is possible to regulate ROS level in pancreatic cancer cells. Based on previous reports, OSGIN1 is an oxidative stress response protein regulated by p53 [32] and induced by DNA damage [31]. Under oxidative stress, OSGIN1 is highly expressed and translocated into mitochondria for the involvement of the mitochondrial events [32]. HMOX1 belongs to a large family of stress proteins whose expression occurs in response to various environmental stimulations [40]. The present results strongly suggested that the functions of RPL10 in mitochondria are closely related to maintenance of ROS homeostasis in the cells, particularly for cancer cells where a higher level of ROS is usually maintained.

### 2.5 RPL10 involved in the regulation of cellular ROS level

**Fig. 4:**
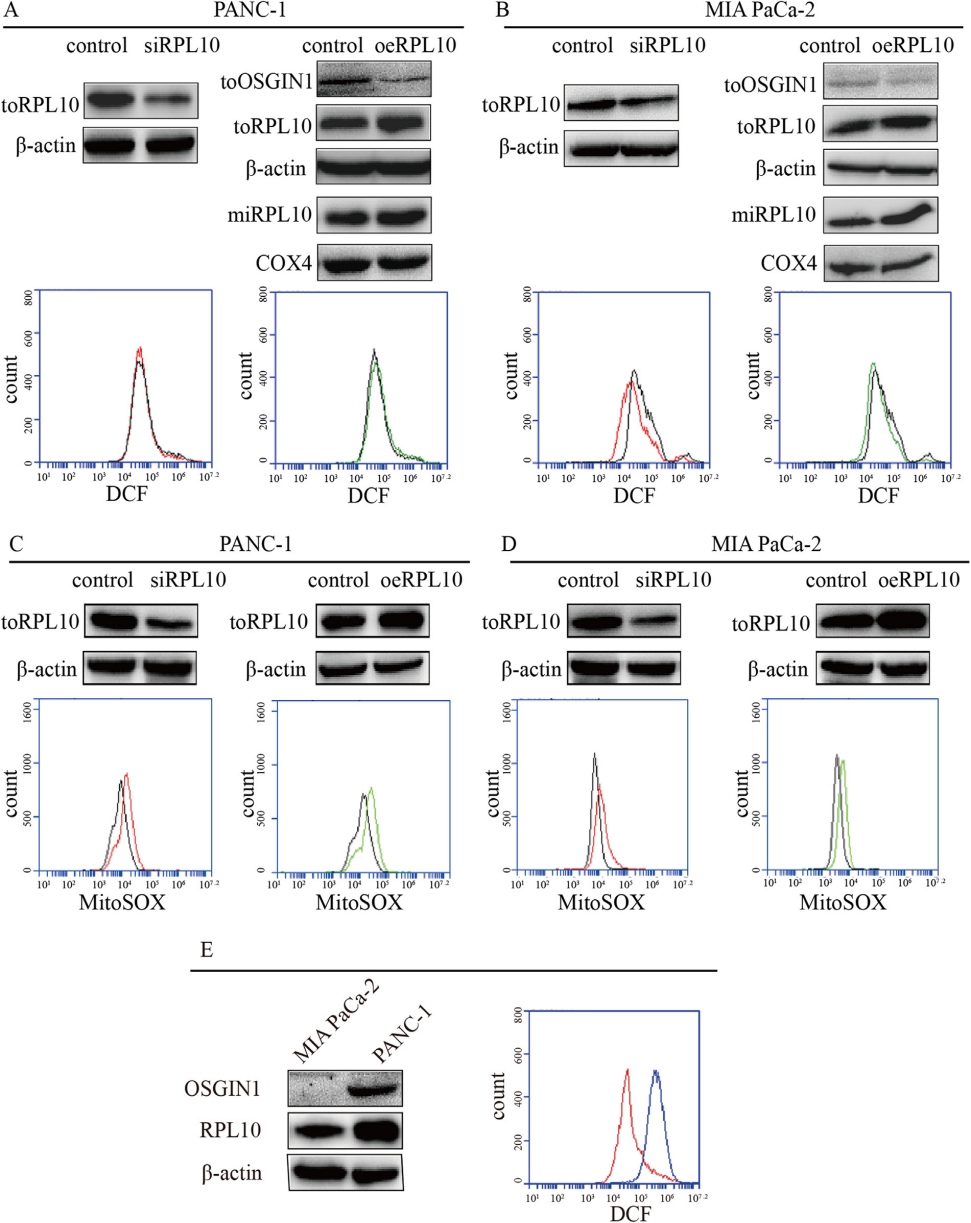
The regulation of ROS by RPL10. (A) Cytoplasmic ROS production after knock-down of RPL10 (siRPL10) and over-expression of RPL10 (oeRPL10) in PANC-1 cells; (B) Cytoplasmic ROS production after knock-down of RPL10 (siRPL10) and over-expression of RPL10 (oeRPL10) in MIA PaCa-2 cells. (C) Mitochondrial ROS production after knock-down of RPL10 (siRPL10) and over-expression of RPL10 (oeRPL10) in PANC-1 cells; (D) Mitochondrial ROS production after knock-down of RPL10 (siRPL10) and over-expression of RPL10 (oeRPL10) in MIA PaCa-2 cells. In the Western blots, toRPL10 and miRPL10 represent RPL10 protein in total and mitochondrial fraction respectively. β-actin and COX4 are reference proteins of total and mitochondrial proteins respectively. In flow cytometry assay, black, red and green lines represent control, siRPL10 and oeRPL10 respectively. DCF and MitoSOX represent respective probes used for detecting cytoplasmic and mitochondrial ROS. (E) Examination of protein expression and ROS level in PANC-1(blue) and MIA PaCa-2 cells (red).

It has been well understood that ROS plays vital roles in tumorigenesis and cell death [41]. Since RPL10 could affect the expression of proteins related to ROS generation, it was conceivable to investigate whether RPL10 participates in the regulation of balancing cellular ROS level. Subsequently, the ROS levels in cytoplasma and mitochondria were determined with DCFH-DA and MitoSOX probes respectively by flow cytometry [42], [43], [44] after knock-down and over-expression of RPL10. In PANC-1 cells, cytoplasmic ROS level showed slight difference compared to control when RPL10 was knocked-down (Fig. 4A), whereas mitochondrial ROS level significantly increased (Fig. 4C) accompanying an increase of OSGIN1 protein (Fig. 3C). When RPL10 was over-expressed, cytoplasmic ROS level was similar to control (Fig. 4A), whereas mitochondrial ROS level clearly increased (Fig. 4C) with a decrease of OSGIN1 expression (Fig. 4A). In MIA PaCa-2 cells, the situation was different. Regardless of knock-down or over-expression of RPL10, cytoplasmic ROS level remarkably decreased (Fig. 4B), whereas mitochondrial ROS level obviously increased (Fig. 4D). The expression difference of OSGIN1 was similar to that in PANC-1 cells (Fig. 3C and Fig. 4B). RPL10 protein in mitochondria showed similar pattern to that in total protein in two cells regardless of its knock-down or over-expression. This significant difference between two cell lines could be due to the background levels of RPL10, OSGIN1 and ROS under physiological conditions. Although both cell lines were derived from pancreatic cancer patients, their original location and disease stage were different. When background expression was examined, it was found that RPL10 and OSGIN1 in MIA PaCa-2 cells were far lower than that in PANC-1 cells (Fig. 4E). Meanwhile, the basal level of ROS in MIA PaCa-2 cells was also lower. The difference of mitochondrial ROS level from RPL10 alteration strongly indicated a regulatory role of RPL10 on ROS level in mitochondria, suggesting that a proper expression of RPL10 is essential for maintaining ROS level in pancreatic cancer cells. These results also indicated that the original redox state might affect the proteins and their signaling pathways to regulate ROS level and consequent cellular fate.

To address the variation of RPL10 with ROS level, exogenously addition of H_2_O_2_ and NAC was performed. When NAC was supplemented to cell cultures, ROS level and the expression of RPL10 decreased in both cell lines (Fig. S2A and Fig. S2B). On the other hand, hydrogen peroxide increased ROS level and decreased the expression of RPL10 in both cell lines (Fig. S2A and Fig. S2B), in which the level of RPL10 seems critical on balancing ROS generation and the resulting oxidative state. Therefore, we reason that RPL10 could serve as a ROS regulator in response to cellular ROS level to direct cellular processes and the final fate of the cells. When ROS level was increased in the cells, the expression of RPL10 would be increased to prevent the production of more ROS. Similarly, when ROS level was decreased, the expression of RPL10 would be decreased to allow the generation of more ROS.

### 2.6 Knock-down of RPL10 arrests cell cycle at S-phase in PANC-1 cells

To examine the phenotype for the involvement of RPL10, cell cycles were determined by flow cytometry assay when RPL10 was knocked-down or over-expressed in PANC-1 and MIA PaCa-2 cells. While no effects were observed from over-expression of RPL10, knock-down of RPL10 resulted in the arrest of cell cycles at S-phase in PANC-1 cells (Supplementary Fig. S3A), which is consistent to previous report that high expression of OSGIN1 may result in cell cycle arrested in S phase [45]. In MIA PaCa-2 cells, there were no significant effects on cell cycles no matter RPL10 was knocked-down or over-expressed (Supplementary Fig. S3B), which is in accordance to the fact that both knock-down and over-expression of RPL10 could decrease cytoplasmic ROS level in MIA PaCa-2 cells. This also suggested that MIA PaCa-2 cells might be more sensitive to the knock-down of RPL10 and the response to the increase of mitochondrial ROS. Thus, it is highly possible that RPL10 may play a buffering role to maintain ROS level within a limited range in cancer cells. Although more detailed mechanistic study on the involvement of ROS regulation and the relationship with pancreatic cancer progression is required, current evidence clearly supports the roles of RPL10 in the regulation of ROS level in the cancer cells.

## 3 Conclusions

In summary, this study revealed that RPL10 could enter to mitochondria for the regulation of ROS homeostasis in cancer cells. Through participating in ATP production and influencing Complex Ⅰ activity in mitochondria, RPL10 could affect the expression of redox related proteins to regulate ROS balance and cellular processes in pancreatic cancer cells. Given that mitochondrion has its own redox buffering system to regulate ROS level including reduced form of glutathione (GSH) and peroxiredoxins [46], RPL10 could be a new member of this unique buffering network in mitochondria. Besides the extra-ribosomal functions previously reported, the present study provides new insights into the roles of RPL10, particularly in mitochondria, in cancer cells and cancer progression, which could offer a new point of view on the functions of ribosomal proteins and their relationship with cancer progression.

## 4 Materials and methods

### 4.1 Cell culture, antibodies and reagents

Pancreatic cancer cells PANC-1 and MIA PaCa-2 were purchased from KeyGEN Biotech (China). They were cultured in Dulbecco's modified eagle medium (DMEM) with high glucose from KeyGEN Biotech, supplemented with 10% fetal bovine serum (Biological Industries, Israel), and 100 U ml^−1^ penicillin and 100 μg ml^−1^ streptomycin at 37 ℃, 5% CO_2_. These antibodies were purchased from indicated companies: rabbit anti-RPL10 (Santa Cruz Biotechnology, USA, 1:1000), rabbit anti-β-actin (Proteintech, USA, 1:2000), rabbit anti-OSGIN1 (Abcam, UK, 1:1000), rabbit anti-HMOX1 (Proteintech, USA, 1:1000), rabbit anti-COX4 (Abcam, UK, 1:1000), rabbit anti-LaminB1 (Abcam, UK, 1:1000). Hydrogen peroxide (H_2_O_2_) and N-acetylcysteine (NAC) were purchased from Sinopharm Chemical Reagent Company (China) and Beyotime Biotechnology (China) respectively.

### 4.2 Preparation of extracts of total proteins, mitochondrial, nuclear and cytoplasmic proteins

The cells were collected, washed twice with PBS and lysed with RIPA buffer (KeyGEN Biotech, China) added with protease inhibitor cocktail (KeyGEN Biotech, China). The lysates were centrifuged at 12,000 ×*g* for 10 min at 4 ℃ and the supernatant was collected as the total protein. The mitochondrial, nuclear and cytoplasmic proteins were prepared strictly according to the Cell Mitochondria Isolation Kit (Beyotime Biotechnology, China) and the Nuclear and Cytoplasmic Protein Extraction Kit (Beyotime Biotechnology, China) with about 10^7^ cells. The protein concentration was determined with a BCA assay (Generay Biotech, China).

### 4.3 RPL10-GFP and RPL10 expression vector construction

To construct the expression plasmid pd1EGFP-N1-RPL10 for RPL10- GFP fusion protein, *Eco*RI and *Bam*HI enzyme sites were chosen. The primer sequences were: 5′-GAATTCATGGGCCGCCGCCCCGCCCGTTGTT-3′ and 5′-GGATCCTGAGTGCAGGGCCCGCCACTTGTCC-3′. Correct fragment was ligated into vector pd1EGFP-N1 (TaKaRa, Japan). RPL10 fragment was cloned into pcDNA3.1 with BamH I and Xho I restriction sites to obtain pcDNA3.1-RPL10 vector.

### 4.4 Transfection

The siRNA for RPL10 was synthesized by Biomics Biotech (China). The sequence is: 5′-GCAUCAACAAGAUGUUGUCdTdT-3′. Cells were seeded in 6-well plates or 10 cm dishes. After 24 h, the siRNA was transfected using Lipofectamine 2000 (Invitrogen, USA) at 30 nM concentration. The vectors for overexpressing RPL10 were transfected at 25 μg concentration. Cells were harvested after 48 h for protein extraction and other detections.

### 4.5 Dual-luciferase reporter assay

The 2000 bp upstream sequences from coding genes fam213b, adamts16 and wt-1 were amplified from the cDNA library of PANC-1 cells and cloned into vector pGL4.17 respectively to obtain pGL4.17-F, pGL4.17-A and pGL4.17-W vectors. After 48 h transfection of pcDNA3.1-rpl10, cells were subsequently transfected with pGL4.17-F/pGL4.74, pGL4.17-A/PGL4.74 and pGL4.17-W/PGL4.74 vectors respectively. pGL4.17/pGL4.74 was used as a control. After 48 h, the cells were collected. The luciferase activity was detected according to the Dual-Luciferase Reporter Assay System (Promega, USA) with Luminometer.

### 4.6 Determination of ATP concentration

Following knock-down of RPL10, PANC-1 and MIA PaCa-2 cells were collected and lysed. The lysates were centrifuged at 12,000 ×*g* for 10 min at 4 ℃ and the supernatant was collected for ATP detection. The ATP concentration was detected according to the ATP assay kit from Beyotime Biotechnology (China). 0.1, 0.5, 1, 5, 10 μM concentrations of the standard were used for generating a standard curve. The ATP detection reagent was diluted 10-fold, and 160 μL of which was mixed with 40 μL samples in 96-well plate. The mixture was detected by Luminometer and the concentration of ATP was calculated according to the standard curve.

### 4.7 Determination of mitochondrial Complex Ⅰ-Ⅳ activity

Cells were collected and washed twice with PBS. The mitochondrial Complex Ⅰ-Ⅳ activity was determined following the Mitochondrial Complex Ⅰ-Ⅳ Activity Assay Kit (Comin Biotechnology, Suzhou, China) with 10 μg mitochondrial proteins. The reaction was prepared according to the instructions from the manufacturer. All reactions were maintained at 37 ℃ for 5 min. The absorbance at different wavelengths at 0 min (A0) and 5 min (A1) was measured with a Luminometer. The complex activity was calculated according to the manuals from the manufacturer.

### 4.8 Determination of intracellular ROS level

Cells were collected and washed twice with PBS. The ROS levels in cytoplasma and mitochondria were assayed with Reactive Oxygen Species Assay Kit (DCF, Beyotime Biotechnology, China) [43] and MitoSOX red mitochondrial superoxide indicator (Invitrogen, USA) [44]. DCFH-DA was diluted to **10μmol/L** with cell medium and loaded into cells for 30 min at 37 ℃. MitoSOX red mitochondrial superoxide indicator was dissolved with DMSO and diluted to 2.5 μM with PBS when loaded into cells for 30 min at 37 ℃. The samples were washed three times with cell culture medium without FBS at 1500 rpm for 5 min each time. Then, the samples were detected with flow cytometry. Ten thousand cells were analyzed every time.

### 4.9 CHIP-Sequencing assay

PANC-1 cells were grown in 15 cm dishes. Approximately 10^8^ cells were collected for CHIP-Seq. and sequencing libraries were prepared following the instruction of EZ-Magna CHIP™ A/G Chromatin Immunoprecipitation Kit (Millipore, USA). The cells were fixed with 1% formaldehyde diluted with growth media for 10 min. The nuclear extracts were prepared and lysed with Nuclear Lysis Buffer. The nuclear lysate was sonicated on wet ice to obtain cross-linked DNA to be 200–500 bp. The standard sonication product was immunoprecipitated with the antibody for RPL10/DNA. Then, cross-links of protein/DNA were treated with Proteinase K to free DNA. The DNA collection was purified with spin columns. Final DNA sample was used as microarray analysis library. The library was sequenced on Illumina HiSeq. 2000 (BGI-Shenzhen, China). Sequence reads were mapped to human reference sequence (GRCh38/hg38). The results were analyzed with UCSC Genome Browser.

### 4.10 Transcriptome sequencing

Transcription-sequencing was performed with PANC-1 cells after RPL10 was knocked-down. The silencing efficiency was about 60% and samples were analyzed by Novogene, China. Total RNA was prepared strictly according to the instructions. Sequencing libraries were generated using NEBNext® Ultra TM RNA Library Prep Kit for Illumina® (NEB, USA). Poly-T oligo-attached magnetic beads were used to purify mRNA. First cDNA strand was prepared with random hexamer primer and M-MuLV Reverse Transcriptase (RNase H^-^). Second strand was subsequently performed with DNA Polymerase and RNase H. After DNA modification, AMPure XP system (Beckman Coulter, Beverly, USA) was used to select the cDNA fragments of preferentially 150–200 bp. Then, adapter-ligation and PCR were performed. Finally, the PCR products were purified and the library quality was assessed on Agilent Bioanalyzer 2100 system. The libraries were sequenced on Illumina HiSeq platform. Sequence reads were referred to GRCh38/hg38.

### 4.11 GFP reporter imaging with confocal laser scanning microscope

PANC-1 cells were seated on confocal laser dishes and transfected with pd1EGFP-N1-RPL10 and pd1EGFP-N1 vectors respectively. 100 nM MitoTracker red staining reagent (KeyGEN Biotech, China) was pre-heated at 37 ℃ before added into the cells. After 48 h transfection, the cells were washed with pre-heated cell media and incubated with MitoTracker red staining reagent at 37 ℃ for 30 min. Then, pre-heated PBS was used to wash the cells for three times. Finally, the cells were examined with confocal laser scanning microscope (CLSM, Olympus FV1000).

### 4.12 Western blotting

Aliquots of proteins (30 μg) were separated on 12% sodium dodecyl sulfate-polyacrylamide gels (SDS-PAGE), and transferred to a 0.45 µm PVDF membrane. The membranes were blocked for 2 h with 5% non-fat milk in Tris-buffered saline (TBS) plus 0.1% Tween-20 (TBST). Then, the membranes were incubated with corresponding antibody diluted in blocking buffer at 4 ℃ overnight. After three 10-min washes with TBST, membranes were incubated at RT for 2 h with appropriate second antibody. Then, three 10 min washes were performed with TBST again. The auto radiographic intensity of each sample was visualized with ECL (Millipore, USA).

### 4.13 Statistical analysis

The statistical significance of comparisons in two groups was analyzed with Student's *t*-test. P values of less than 0.05 were thought to be significant. The data were analyzed with GraphPad Prism 5.
