# Supplementary material for: Ribosomal protein L10 in mitochondria serves as a regulator for ROS level in pancreatic cancer cells
Source: Redox Biol. 2018 Aug 24;19:158–65. doi: 10.1016/j.redox.2018.08.016 (PMC6122146; doi:10.1016/j.redox.2018.08.016)
Supplement: Supplementary file 1 — Supplementary material [file mmc1.docx]

**Supplementary Materials for:**

**Ribosomal Protein L10 in Mitochondria Serves as a**

**Regulator for ROS Level in Pancreatic Cancer Cells**

Jun Yang^1,a^, Zongmeng Chen^1,a^, Nan Liu^a^ and Yijun Chen^*a^

**Supplementary Materials**

Table 1. Significantly different genes with a padj< 10^-4^ from the transcriptsome sequencing analysis

| GeneName | Log_2_FoldChange^a^ | padj^b^ | GeneType | GeneDescription |
| --- | --- | --- | --- | --- |
| FP236383.10 | -5.910578927 | 2.12E-25 | miRNA | NA |
| HMOX1 | 3.832621396 | 3.27E-12 | protein_coding | heme_oxygenase_1 |
| OSGIN1 | 3.598263875 | 5.85E-10 | protein_coding | oxidative_stress_induced_growth_inhibitor_1 |
| SPNS2 | 3.184695906 | 1.20E-08 | protein_coding | spinster_homolog_2_(Drosophila) |
| RPL10 | -2.759861577 | 1.58E-06 | protein_coding | ribosomal_protein_L10 |
| DHRS9 | 2.995416325 | 1.59E-06 | protein_coding | dehydrogenase/reductase_(SDR_family)_member_9 |
| ATF3 | 2.623921568 | 1.03E-05 | protein_coding | activating_transcription_factor_3 |
| PPP1R15A | 2.46923802 | 5.28E-05 | protein_coding | protein_phosphatase_1_regulatory_subunit_15A |
| TIPARP | 2.451971027 | 5.28E-05 | protein_coding | TCDD-inducible_poly(ADP-ribose)_polymerase |

^a^The log_2_ value of ratio of treated group-Expression to control group-Expression; ^b^padj- adjusted p-value


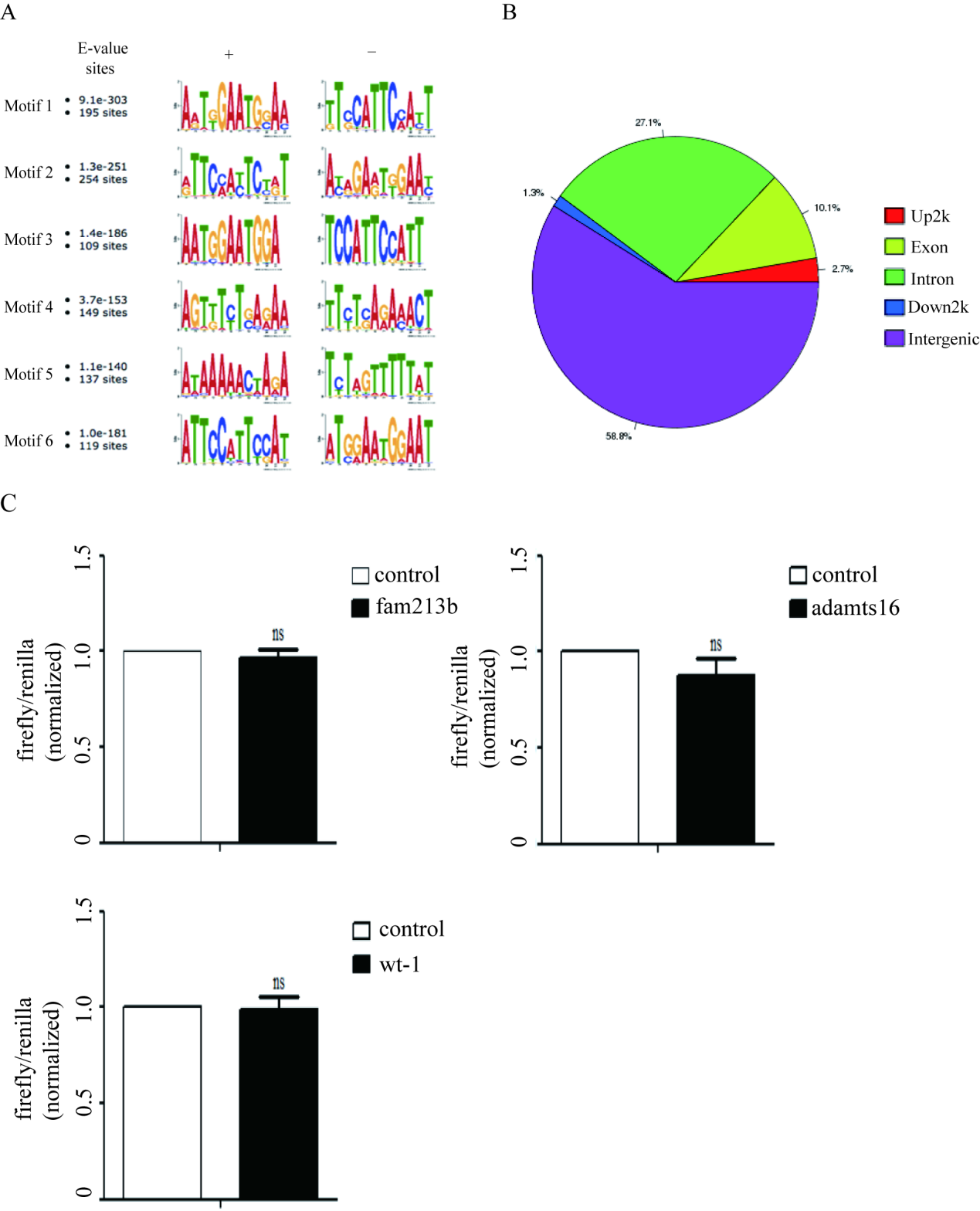


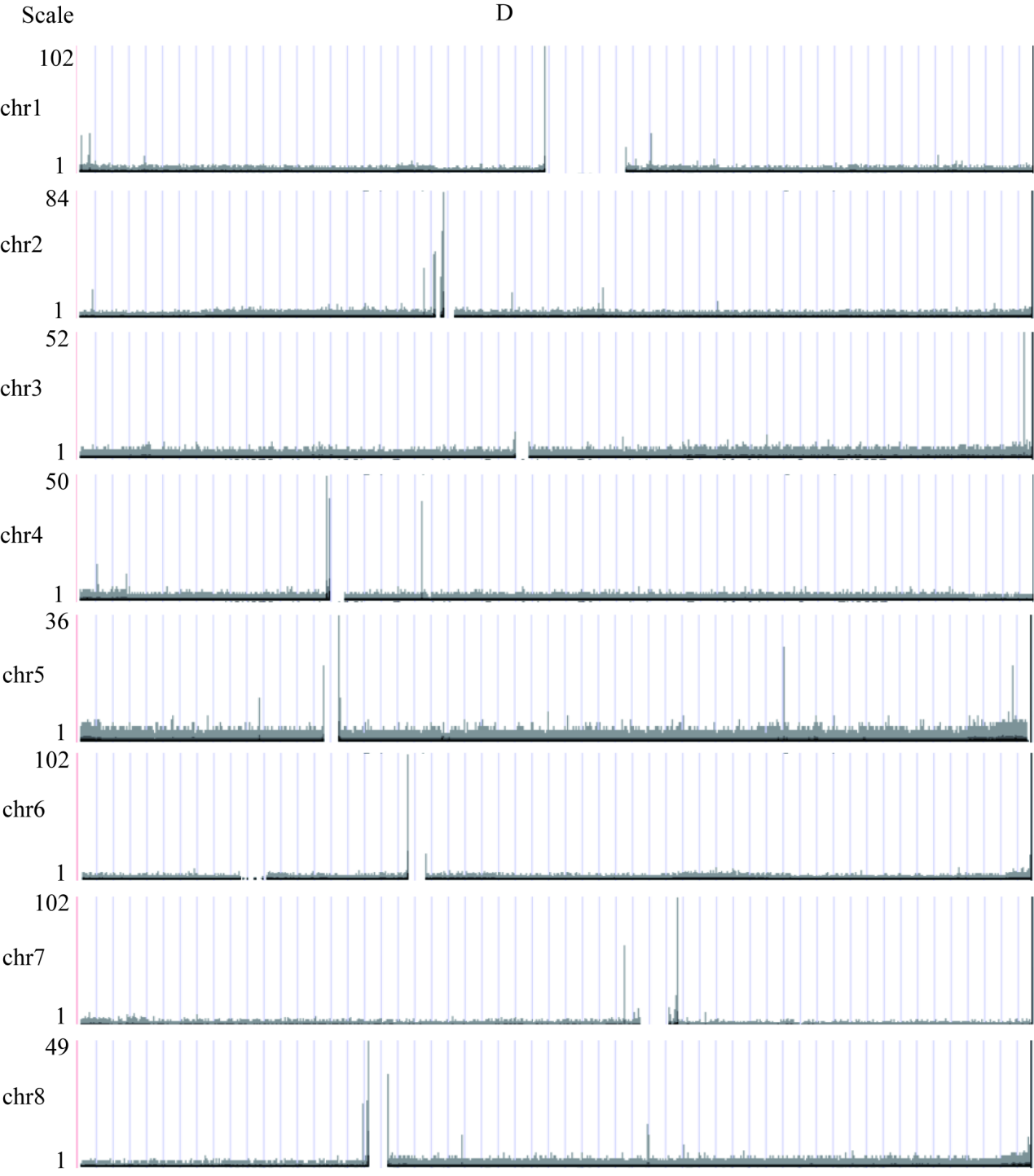


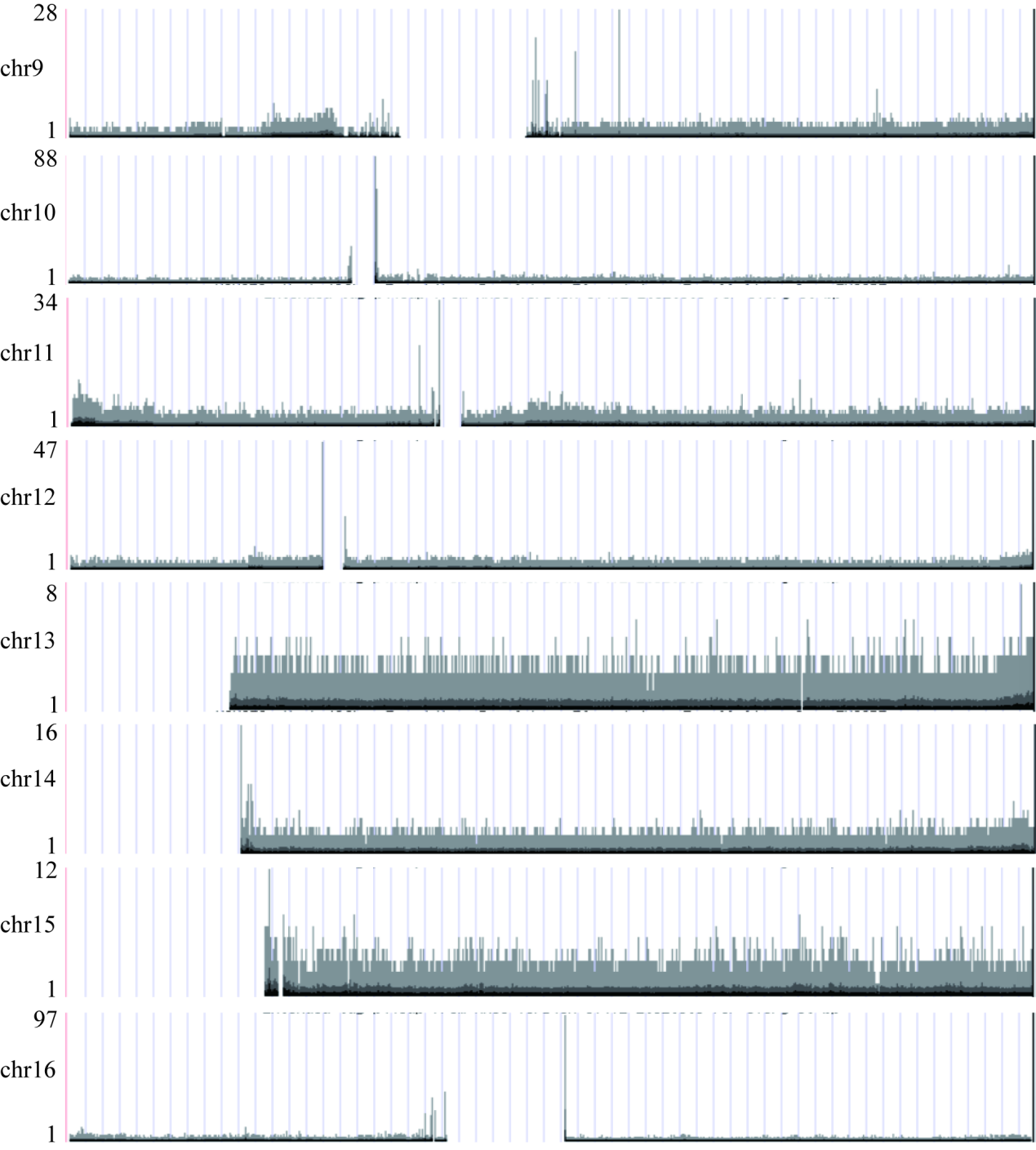


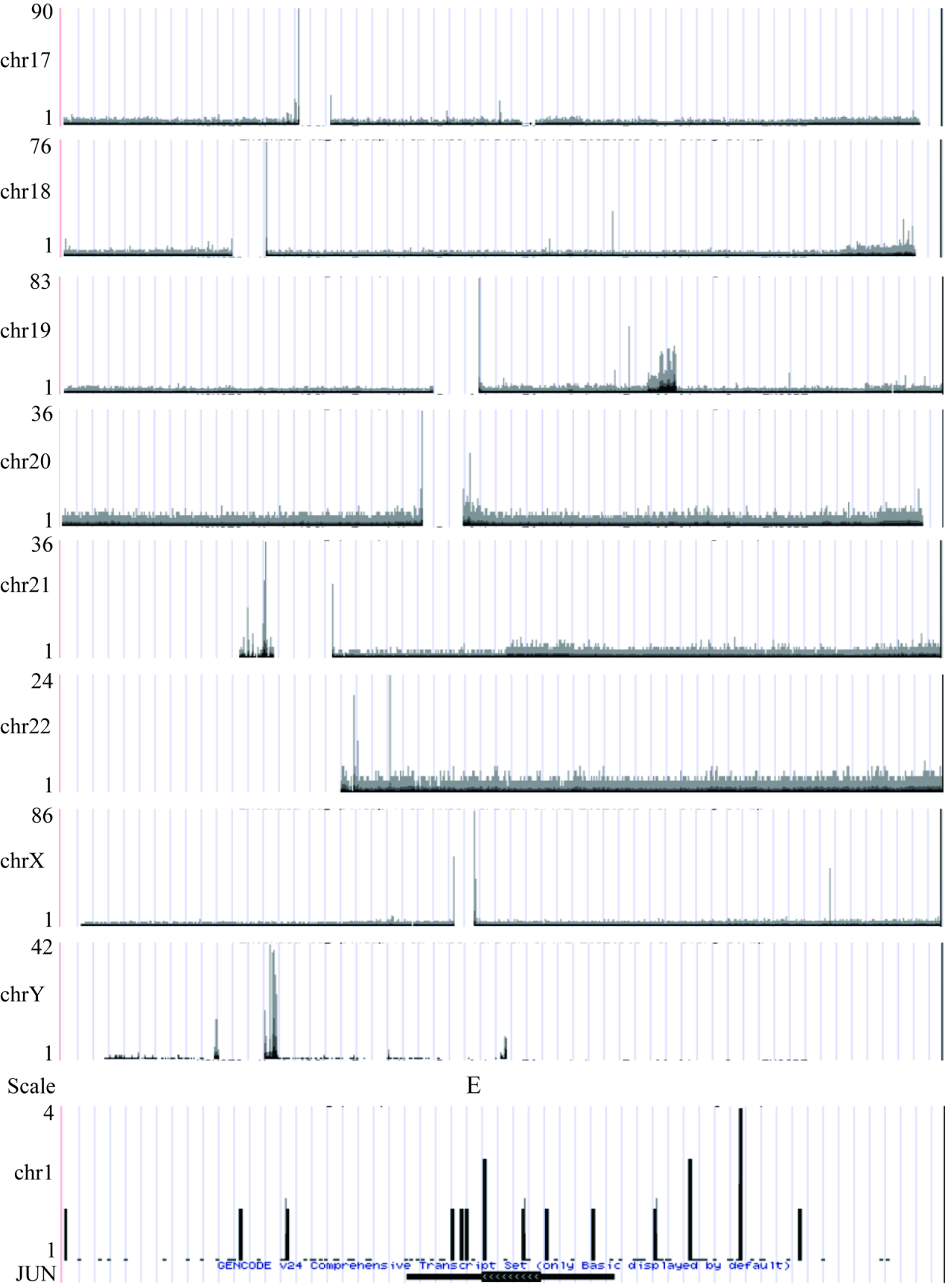


Figure S1. Exclusion of RPL10 as a transcription factor. (A) Motifs obtained from CHIP-Seq. E-value is the statistical significance of the motif and sites are the number contributing to the construction of the motif. “+” means the sequence in the training set and “-” means the reverse complement of the training set sequence. T is representative of U in the RNA. (B) Genome locations of the motifs. All peaks were classified based on the location on the genome: upstream (Up2k), extron, intron, downstream (Down2k) and intergenic regions. (C) Luciferase reporter detection. Three genes were fam213b, adamts16 and wt-1. Firefly luciferase activity was normalized to Renilla activity. “ns” p >0.05. (D) Signals enriched in all chromosomes. In the signal maps, scale represents the signals in the chromosomes. (E) Signals enriched in c-jun gene.


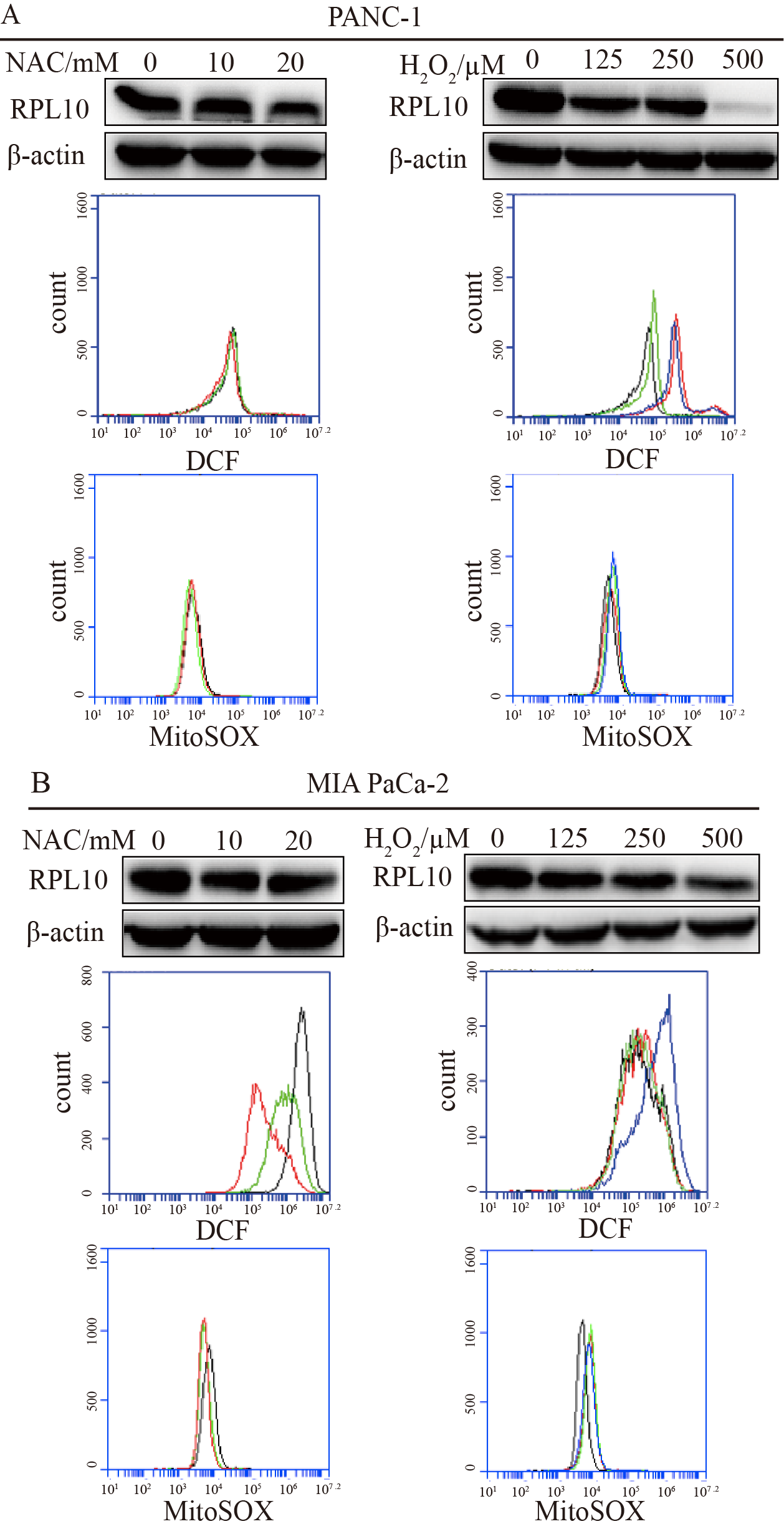


Figure S2. Effects of NAC and H_2_O_2_ on RPL10 expression. (A) Effects of NAC and H_2_O_2_ on RPL10 expression in PANC-1 cells; (B) Effects of NAC and H_2_O_2_ on RPL10 expression in MIA PaCa-2cells. NAC concentrations are 0mM (black), 10mM (green) and 20mM (red) and H_2_O_2_ concentrations are 0μM (black), 125μM (red), 250μM (green) and 500μM (blue). DCF and MitoSOX represent respective probes used for detecting cytoplasmic and mitochondrial ROS.


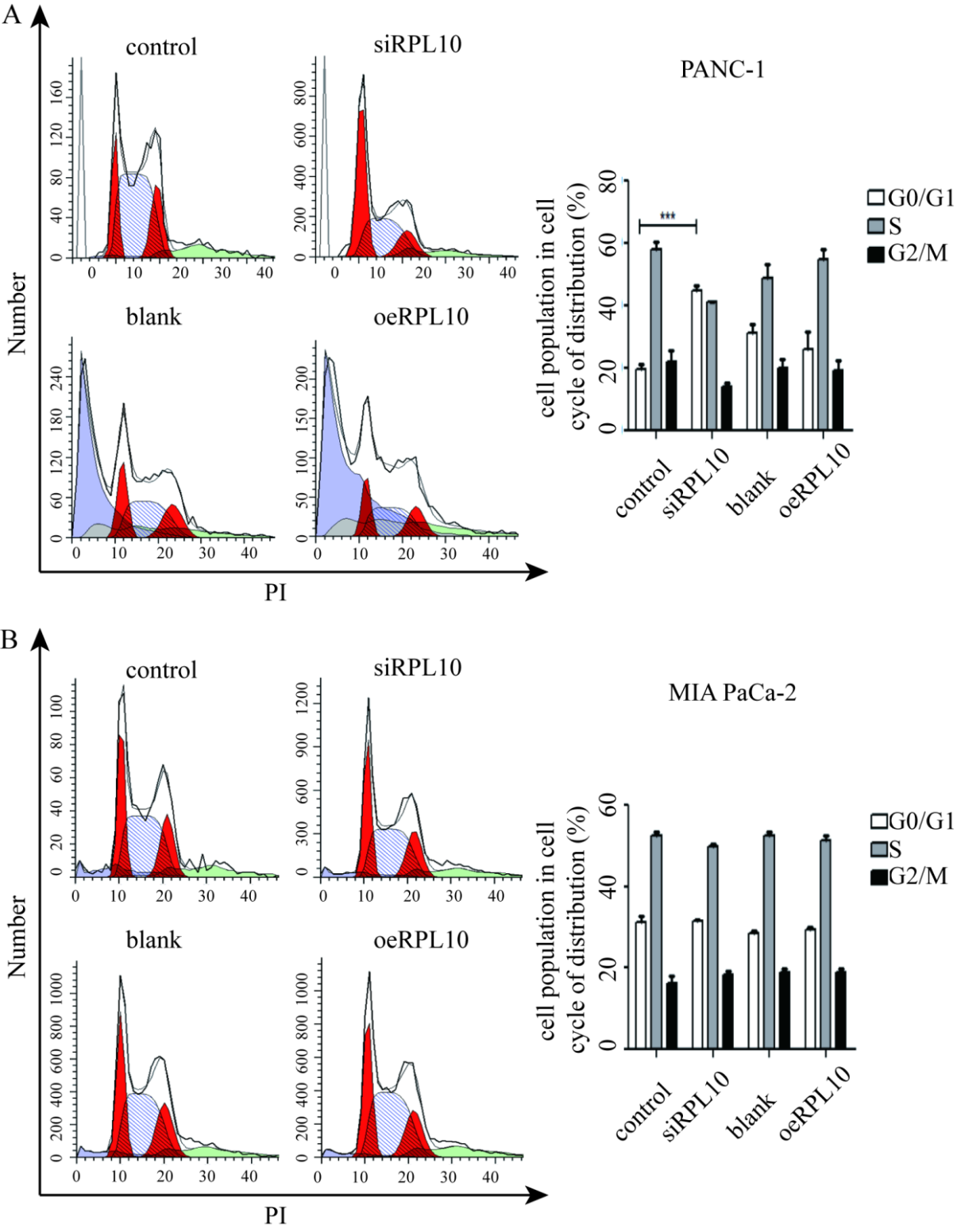


Figure S3. Effects on cell cycles after knock-down and over-expression of RPL10. (A) Effects on PANC-1 cell cycles. (B) Effects on MIA PaCa-2 cell cycles. Control is in contrast to siRPL10, and blank is in contrast to oeRPL10. In flow cytometry assay, X-axis and Y-axis are the channels for PI staining and cell number in each phase of cell cycles respectively. G0/G1, S and G2/M represent different phases of cell cycles. Three independent experiments were performed. *** P< 0.001.
